# Profile of Hypothyroidism in Down’s Syndrome

**DOI:** 10.4274/Jcrpe.884

**Published:** 2013-05-30

**Authors:** Ayşe Nurcan Cebeci, Ayla Güven, Metin Yıldız

**Affiliations:** 1 İstanbul Medeniyet University, Göztepe Education and Research Hospital, Department of Pediatric Endocrinology, İstanbul, Turkey

**Keywords:** Down’s syndrome, hypothyroidism, thyroid dysgenesis

## Abstract

**Objective:** Although the association between Down’s syndrome (DS) and thyroid dysfunction is well recognized, the cause of this condition is not known.

**Methods:** Hospital records of patients with DS and hypothyroidism referred to our clinic were retrospectively reviewed. Initial thyroid hormone and thyrotropin (TSH) levels, age at admission, initial anthropometric measurements, age at the beginning of therapy, initial L-thyroxine (L-T4) doses, time to normalization of the thyroid function tests, and L-T4 dose at last visit were recorded. Thyroid ultrasound imaging was used to measure the size of the gland. Descriptive data were expressed as mean±SD values. Skewed data were shown as median and interquartile ranges (IQR).

**Results:** There were 62 patients with DS (32 male and 30 female). Median TSH level at the first visit was 10.40 (19.4) µIU/mL and median free T4 level was 1.18 (0.43) ng/dL. There was no statistical difference in terms of age, hormone and antibody levels, thyroid volume and L-T4 doses between boys and girls. Thyroid volumes of 54 patients were measured. Only nine of these patients had a normal-sized thyroid gland. Median total thyroid volume was 0.89 (2.07) mL. Thyroid volume was negatively correlated to L-T4 dose at last visit (p=0.006, r=-0.387).

**Conclusions:** We found a high prevalence of thyroid dysgenesis in patients with DS and hypothyroidism. This association has not been reported before. Further studies investigating the thyroid gland size in these patients need to be performed to confirm the results.

**Conflict of interest:**None declared.

## INTRODUCTION

The association between Down’s syndrome (DS) and thyroid disorders is well recognized. Patients with DS have an increased prevalence of both congenital hypothyroidism (CH) and acquired thyroid dysfunction (1,2,3). Former studies demonstrated that the frequency of CH in these patients was 28 times higher than in the general population (2). Mild plasma thyrotropin (TSH) elevation with normal thyroxine (T4) levels is the most commonly seen pattern of thyroid dysfunction in DS (4,5,6,7). Gibson et al (7) measured TSH and T4 levels in 122 children with DS aged 6-14 years, repeated the measurements four to six years later in 103 adolescents aged 10-20 years, and reported that these biochemical deviations decreased with age - seventy percent of individuals with subclinical hypothyroidism in the first test had become normal in the second one. On the other hand, a recent longitudinal study has demonstrated that the prevalence of normal thyroid function in patients with DS significantly decreased from 90.8% to 41.7% throughout a 10-year follow-up period (8). These latter authors have concluded that patients with DS should be carefully followed annually. The prevalence of thyroid dysfunction in DS might be even higher than previously reported.

To date, published cross-sectional and longitudinal series in children with DS have covered issues on the frequency and the natural course of hypothyroidism. Yet, the cause of neonatal hypothyroidism in this particular syndrome remains unclear; most patients have normal thyroid scans excluding thyroid agenesis or ectopic thyroid tissue (2,9,10,11). The absence of goiter suggests that it is not caused by dyshormonogenesis (2,10,12).

In this study, we analyzed the characteristics of children with DS receiving L-T4 treatment, aiming to present data for a better understanding of the underlying etiology. 

## METHODS

Hospital records of patients with DS receiving L-T4 treatment were retrospectively reviewed. Patients with CH or acquired hypothyroidism and with abnormal initial thyroid function tests were included in the series, while patients who discontinued treatment and those who had not been followed regularly were excluded.

All patients with abnormal thyroid function test results had been referred to our pediatric endocrinology outpatient clinic. Definition of thyroid dysfunction was based on elevated TSH levels. TSH was accepted as elevated if serum TSH level was above 20, 10, and 5 mU/L in DS patients aged from birth to one week, from eight days to one month, and those older than one month, respectively. Subclinical hypothyroidism was considered in cases with a TSH level between 5-10 mU/L and normal free T4 (fT4) level. A diagnosis of overt hypothyroidism was made in infants/children with low fT4 levels and TSH levels greater than 10.1 mU/L.

Since this study was a retrospective study, all requisite information was not available for all cases. Of available cases, initial fT4 and TSH levels, age at admission, initial anthropometric measurements, and ages of both parents at birth were recorded. Age at the beginning of therapy, initial L-T4 doses, time to normalization of the thyroid function tests, and L-T4 dose at last visit were also recorded.

Thyroid ultrasound imaging was used to measure the size of the gland. The height, width, and depth of each lobe were measured and multiplied. The obtained result was then multiplied by a correction factor, which is 0.479 according to the recommendation of the World Health Organization (13). The calculated volume was then compared to the normal thyroid gland volume values of Turkish healthy children reported by Kurtoglu et al (14). Hormone determinations were made by the chemiluminescence method using Beckman Counter, DX1800 Access Immunoassay Systems. The data for each sex were treated separately and were compared.

Statistical analysis was performed by using SPSS 15.0 (SPSS, Chicago, IL) statistical software. Shapiro-Wilk test was used to test the normality of the data. Descriptive data were expressed as mean±SD values. Skewed data were shown as median and interquartile range (IQR). Patients with CH and acquired hypothyroidism were compared regarding their initial fT4 levels, L-T4 doses at last visit, and parental ages by student’s t test. Kruskal-Wallis nonparametric analysis of variance test followed by Mann-Whitney U test were used in the analysis of data on initial TSH levels, age at admission, initial anthropometric measurements, age at the beginning of therapy, initial L-T4 doses, time to normalization of the thyroid function tests, size of each thyroid lobe by gender and thyroid volume. The Pearson’s correlation coefficient was used to evaluate the relationships between variables. For all tests, a p-value of less than 0.05 was accepted as statistically significant.

## RESULTS

There were 62 eligible patients with DS (32 male, 30 female). Diagnosis of DS was confirmed by genetic analyses in 20 of the patients. The remaining 42 patients were diagnosed by clinical findings. Median age at admission was 14.5 months for the boys and 33.0 months for the girls (min 0.4 - max 209 months). Mean maternal age at the time of birth was 32.8±6.9 years (min 19 - max 45 years), mean paternal age at the time of birth was 36.0±6.9 years (min 25 - max 54 years). The patients have been followed for a median period of 21 months (min 2 - max 138 months).

Only 10 patients in the series had symptoms on admission. Seven of these had prolonged neonatal hyperbilirubinemia, two had constipation, and one patient had inguinal hernia. All the remaining patients were referred due to elevated TSH levels detected either by neonatal screening (in 15 patients) or by routine screening of DS.

Median age at the beginning of L-T4 treatment was 14.5 months (min 0.27 - max 209 months) for males and 33.0 months (min 0.4 - max 209 months) for girls. Median (IQR) time to normalization of the thyroid function tests was 21.0 (15) days. Median (IQR) initial L-T4 dose was 25 µg/day (min 25 - max 100 µg/day).

Anti-thyroglobulin and anti-thyroid peroxidase levels were measured in 14 patients who were admitted after infancy. Antibodies were found to be positive in 8 of these patients (5 male, 3 female). A male patient who was admitted at age 15 months and who had initially been diagnosed as CH and thyroid hypoplasia developed encephalopathy at age 4.25 years. He was subsequently diagnosed as Hashimoto encephalopathy. A repeat ultrasound imaging in this patient revealed thyroid hypoplasia with heterogeneous parenchyma.

Median (IQR) TSH level at first visit was 10.40 (19.4) µIU/mL (N=0.85-6.5, min 5.6 - max 100 µIU/mL). Median (IQR) fT4 level was 1.18 (0.43) ng/dL (N=0.93-1.7, min 0.50 - max 1.7 ng/dL). The results were compared between boys and girls. There was no statistical difference in terms of age, hormone and antibody levels, thyroid volume and L-T4 doses between these two groups (Table 1).

In the correlation analyses, TSH levels were found to be negatively correlated with fT4 levels (r=-0.331, p=0.020) and with age (r=-0.270, p=0.046) and positively correlated with initial doses of the treatment (r=0.274, p=0.045). TSH levels were found not to be correlated with either thyroid volume or time to normalization of the thyroid function tests.

Thyroid volumes of 54 patients were measured using ultrasound. No patient had thyroid agenesis. Median (IQR) thyroid volume was 0.89 (2.07) mL (min 0.08 - max 8.40 mL). Of 54 patients with a thyroid ultrasound imaging, 45 patients (83%; 23 girls, 25 boys) were found to have hypoplasia of the gland. Of 41 patients with CH, thyroid volume had been measured by ultrasonography in 37, and in 35 of these patients (95%), hypoplasia was detected. Of 21 patients with acquired hypothyroidism, 17 had thyroid measurement via ultrasonography, and 10 of these patients (59%) had thyroid hypoplasia. Median (IQR) thyroid volume was 0.72 (0.58) mL in patients with CH and 2.9 (3.16) mL in patients with acquired hypothyroidism. Thyroid volumes in patients with CH were significantly smaller than those in patients with acquired hypothyroidism (p<0.001).

Interestingly, thyroid volume was found to be normal for age in only 9 patients; in 6 of these, ultrasound imaging was compatible with autoimmune thyroiditis. As expected, thyroid volume was strongly correlated with age at admission (r=0.774, p<0.001), age at the beginning of therapy (r=0.730, p<0.001), height (r=0.742, p<0.001), and weight (r=0.757, p<0.001). Thyroid volume was also found to be positively correlated with initial L-T4 doses (r=0.453, p=0.001) and negatively correlated with L-T4 dose at last visit (r=-0.376, p=0.008).

## DISCUSSION

The results of this retrospective study showed that patients with DS have a high prevalence of CH associated with hypoplasia of the thyroid gland. Transient mild TSH elevation is the most commonly seen thyroid dysfunction in children with DS (4,5,6,7,9). It has been postulated that infants with transient TSH elevation have a higher incidence of congenital malformations than infants with permanent CH (15). It can be hypothesized that newborns with congenital malformations similar to DS suffer perinatal stress, which may lead to TSH elevation. A recent study reported a higher TSH standard deviation score in infants with DS as compared to healthy newborns at neonatal screening and suggested that elevated TSH levels in early life could not predict development of manifest thyroid disease later in childhood (16).

Besides transient thyroid dysfunction, DS is also associated with long-term thyroid dysfunction. Several studies have stated remarkably higher prevalence of persistent primary CH in DS (2,17). Lower T4 concentrations at neonatal screening of DS newborns have been recently reported that could not be explained by prematurity, nonthyroidal illness or iodine exposure (18). In our study, the early median age at the beginning of L-T4 replacement and the high percentage of thyroid dysgenesis suggest that the majority of our cases have CH.

The most remarkable outcome of our study was the high prevalence of thyroid hypoplasia. Ultrasound imaging revealed that more than eighty percent of the patients had a small thyroid gland for their age. Since this study was not a prospective study, more than one radiologist have performed the imaging, excluding the possibility of false reporting. The volumes have been calculated by the authors and not by the radiologists, as explained in detail in the Methods section. It should be noted that we included only the patients with abnormal thyroid function tests in the present study. We evaluated not all patients with DS but those who had been referred to the Pediatric Endocrinology Department with thyroid dysfunction. In a longitudinal study, Tuysuz and Beker (19) showed that the prevalence of CH was 1.8% in Turkish children with DS, while 25.3% of them had compensated hypothyroidism. However, the size of the thyroid gland has not been investigated in that study.

Although the cause of CH in DS remains unclear, most patients have normal thyroid scans excluding athyreosis or an ectopic thyroid gland (2,9). Based on autopsy findings, it was concluded that the fetal growth abnormality of the thyroid gland leads to hormonal dysfunction (20). However, these studies are all old studies using nuclear imaging which cannot possibly identify thyroid hypoplasia. The findings of former studies need to be confirmed with a newer technique such as three-dimensional ultrasound imaging. We suggest that the most common cause of CH in DS is thyroid dysgenesis. Thyroid volume in all patients with definite CH and DS has to be measured and compared to age-matched controls.

Patients with DS are known to be prone to develop autoimmune thyroid disease in later life, which frequently leads to hypothyroidism (3). Fourteen patients had acquired hypothyroidism in our study. Eight of these patients had elevated anti-thyroid antibodies, 6 of them showing chronic inflammation in ultrasound imaging. Increase in anti-thyroid antibody positivity through adulthood, leading to hypothyroidism, has been documented in DS (21,22,23,24). Although autoimmune thyroid disease is mostly seen in females, in our study, five boys and three girls had thyroiditis. Since the numbers were small to compare, no interpretation could be made.

Although the Scottish Down Syndrome Thyroid Screening Group recently suggested that most patients with mildly elevated TSH levels (6-10 mU/L) do not require treatment but only surveillance initially (25), many studies claim the contrary. van Trotsenburg et al (26) demonstrated that L-T4 replacement for the first two years of life could improve psychomotor development and growth in infants with DS. Moreover, it has been recently shown that subclinical hypothyroidism and low-normal fT4 levels in patients with DS may have significant clinical sequelae, such as hypotonia and anemia (27). Since the untreated hypothyroidism may aggravate several of the manifestations associated with DS, it is particularly important that L-T4 treatment be initiated as soon as possible.

One of the limitations of our study is its retrospective design; hence, we could not demonstrate the benefits of treatment on physical or mental development. However, since the prevalence of thyroid dysgenesis is so high in DS, we believe that thyroid hormone replacement should be applied to all children with DS and permanent hypothyroidism. Mean L-T4 dose at last visit was found to have been reduced to 2.6 µg/kg daily, a finding which shows that low-dose treatment is adequate to suppress TSH. Patients who did not require treatment in infancy should regularly be monitored in terms of developing autoimmune thyroid disease.

In conclusion, in this study investigating the possible etiology of hypothyroidism in DS, we found a high prevalence of thyroid dysgenesis in DS patients with permanent thyroid dysfunction. This association has not been reported before, so further studies investigating the thyroid gland size with ultrasound technique need to be performed to confirm our results. We suggest that all patients with DS should be screened for thyroid dysgenesis, and if present, lifelong treatment with L-T4 should immediately be started. 

## Figures and Tables

**Table 1:**
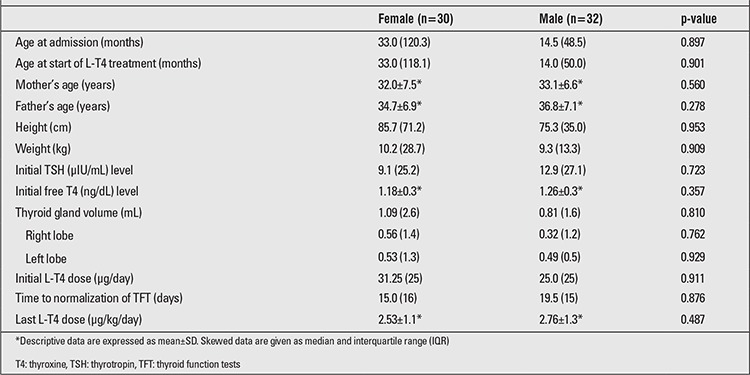
Comparison of demographic and clinical features in female and male patients with Down’s syndrome
